# MURIN: Multimodal Retinal Imaging and Navigated-laser-delivery for dynamic and longitudinal tracking of photodamage in murine models

**DOI:** 10.3389/fopht.2023.1141070

**Published:** 2023-04-06

**Authors:** Jose J. Rico-Jimenez, Joel Jovanovic, Stephanie L. Nolen, Joseph D. Malone, Gopikrishna M. Rao, Edward M. Levine, Yuankai K. Tao

**Affiliations:** ^1^ Department of Biomedical Engineering, Vanderbilt University, Nashville, TN, United States; ^2^ Vanderbilt University Medical Center, Vanderbilt Eye Institute, Nashville, TN, United States; ^3^ Deparment of Ophthalmology and Visual Sciences, Vanderbilt University, Nashville, TN, United States; ^4^ Department of Cell and Developmental Biology, Vanderbilt University, Nashville, TN, United States

**Keywords:** ophthalmic imaging, optical coherence tomography, scanning laser ophthalmoscopy, fluorescence, photodamage

## Abstract

**Introduction:**

Laser-induced photodamage is a robust method for investigating retinal pathologies in small animals. However, aiming of the photocoagulation laser is often limited by manual alignment and lacks real-time feedback on lesion location and severity. Here, we demonstrate MURIN: MUltimodal Retinal Imaging and Navigated-laser-delivery, a multimodality OCT and SLO ophthalmic imaging system with an image-guided scanning laser lesioning module optimized for the murine retina. The proposed system enables targeting of focal and extended area lesions under OCT guidance to benefit visualization of photodamage response and the precision and repeatability of laser lesion models of retinal injury.

**Methods:**

MURIN optics were optimized for simultaneous near-infrared and visible wavelength imaging/laser lesioning. Custom LabView control software was developed to steer the photocoagulation laser and automatically deliver laser pulses to targets-of-interest. *In vivo* retinal imaging was performed in transgenic Müller glia-tdTomato reporter mice (*Rlbp1:CreER; Rosa^ai14^*, 5 animals, 10 eyes) and microglia-GFP/Müller glia-tdTomato reporter mice (*Cx3cr1^GFP^; Rlbp1:CreER; Rosa^ai14^*, 9 animals, 15 eyes) to visualize cellular changes in the retina after laser lesion delivery.

**Results:**

Real-time MURIN imaging concurrent with laser lesioning allowed us to visualize lesion formation dynamics and any corresponding changes in retinal morphology. We observe increasing fluorescence photoconversion on SLO and scattering contrast on OCT. Significant morphological changes are visible on MURIN after high-severity photodamage. OCT cross-sections show the spatial extent of the lesions contract over time from diffusion areas of increased scattering to granular scatterers and corresponding SLO images show a radial pattern surrounding severe focal lesions, which may be a result of a change in Müller cell shape or orientation in response to injury. The inner plexiform layer is distorted and increased RPE thickness and scattering are observed, all of which are confirmed on corresponding hematoxylin and eosin (H&E) histology and differential interference contrast (DIC) microscopy.

**Discussion:**

MURIN as a unique imaging platform that enables combined SLO and OCT imaging with an integrated image-guided laser lesioning module. This technology has clear benefits over existing multimodal imaging and laser lesioning systems by enabling simultaneous multimodal imaging, independent and precise control of Iridex laser pulse parameters and patterns, and real-time OCT and SLO visualization of lesion formation.

## Introduction

Laser lesioning is a robust method for studying retinal photodamage and modeling human pathologies in small animals (1–3). Current methods for inducing photodamage require dedicated slit-lamp integrated laser delivery systems, and outcomes are highly variable because lesion localization and severity are often limited by operator experience (4, 5). The FDA-approved pattern scanning laser (PASCAL) photocoagulator overcomes some of these limitations by implementing preset treatment patterns (6). However, aiming of the treatment area is performed using a slit-lamp or indirect ophthalmoscope, which provides limited visualization of retinal structural changes and no quantitative feedback on injury severity. While multimodal imaging and lesion delivery systems that enable more precise localization of lesions have been demonstrated, state-of-the-art implementations require extended laser dwell times on the retina and preclude real-time visualization of laser delivery (7–9).

Optical coherence tomography (OCT) provides high-resolution volumetric imaging of tissue scattering and scanning laser ophthalmoscopy (SLO) enables visualization of endogenous and exogenous fluorescence contrast (9–12). These optical imaging technologies uniquely enable non-invasive access to *in vivo* cellular and subcellular retinal anatomy without the need to excise tissues. OCT and SLO systems are routinely used for ophthalmic diagnostic imaging in humans and animal models, and a combined OCT and SLO system can provide complementary information about changes in retinal structure and function immediately and at longitudinal time points after injury.

Here, we introduce MURIN: MUltimodal Retinal Imaging and Navigated-laser-delivery, which combines a multimodality OCT and SLO ophthalmic imaging system with an integrated scanning laser lesioning module to enable precise image-guided laser lesioning in the murine retina. We also show real-time fluorescence photoconversion of tdTomato provides high-sensitivity feedback on laser lesion location and retinal scattering measured using OCT enables three-dimensional localization of injury extent and severity. Finally, we leverage machine-learning algorithms to automatically segment retinal photolesions using OCT data, which can be used to track changes in retinal morphology days and weeks post-injury.

## Materials and methods

### System design and optimization

**Figure 1:**
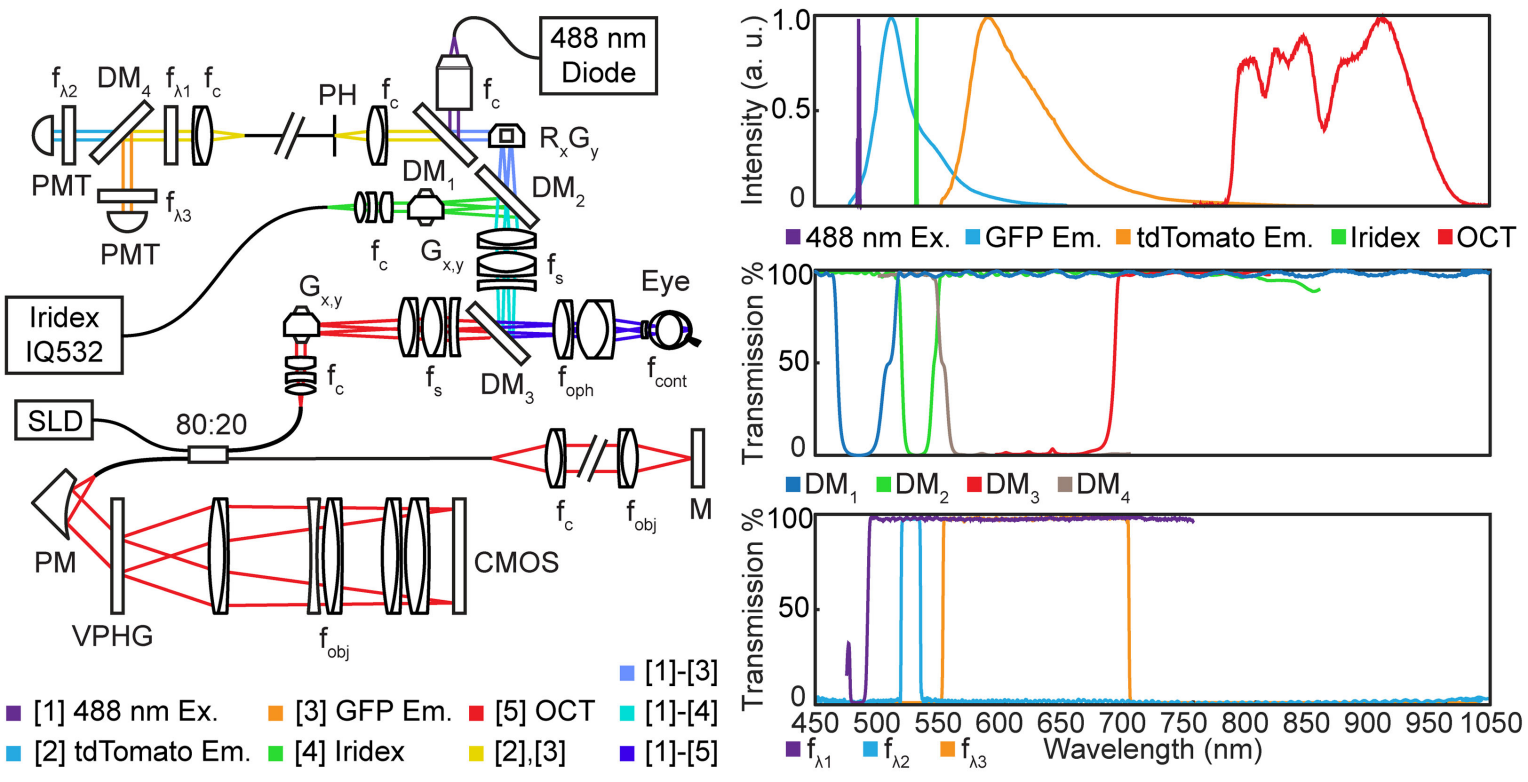
System schematic showing optical components and relays in MURIN. Inset includes spectra for all light sources and fluorophores and transmission spectral for all dichroic mirrors and filters. CMOS, CMOS detector; DM, dichroic mirrors; fλ: spectral filters; f, collimating, objective, and scan lens; G, galvanometers; M, mirror; PM, parabolic mirror; PMT, photomultiplier tube; R, resonant scanner; SLD, super luminescent diode; VPHG, volume phase holographic grating.

**Figure 2:**
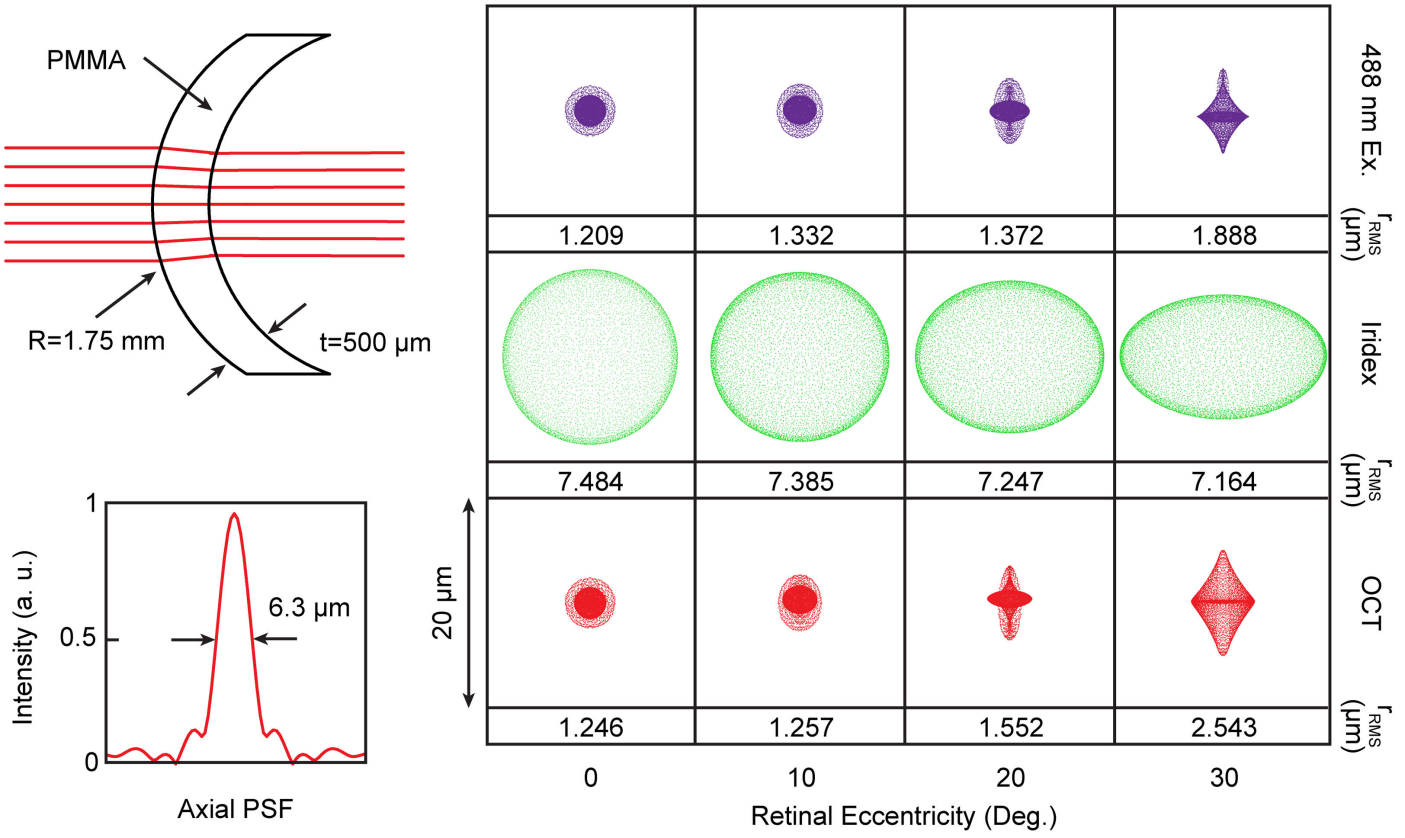
Optical schematic of contact lens and corresponding optimize spot sizes for SLO excitation, lesioning laser, and OCT. Spot sizes are shown up to 30 deg. retinal eccentricity, which corresponds to maximum FOV in the murine eye. Corresponding lateral spot sizes are shown as root-mean-square radii and inset shows OCT axial point-spread-function. Black arrows point to corresponding dimensions (i.e., R - radius of curvature, t - thickness, PMMA - lens material, full width half maximum of axial PSF).

MURIN optics were optimized in ZEMAX for simultaneous near-infrared and visible wavelength imaging/laser lesioning (**Figure 1**). OCT imaging was performed using a custom-designed spectrometer at 875 ± 75 nm (Superlum, M-T-870-HP) with 6.3 µm axial resolution in air and 107 dB signal-to-noise ratio with 1.5 mW at the pupil. SLO imaging used a 488 nm diode with 200 µW at the pupil for simultaneous tdTomato and green fluorescence protein (GFP) excitation. Laser lesioning was performed using a 532 nm photocoagulation laser (IQ532, Iridex) delivered through a multimode fiber (200 µm mode-field diameter). OCT and laser lesioning beam paths were scanned using respective galvanometer pairs and SLO scanning was performed using a modified resonant-galvanometer confocal scan head (Thorlabs). Laser lesioning and SLO beam paths were combined after their respective scanners across a notch dichroic mirror (515-555 nm reflection) and the combined visible wavelength path was combined with the near-infrared OCT path across a 685 nm long-pass dichroic. OCT, SLO, and laser lesioning sources were all collimated and then relayed across a 2x demagnifying telescope with shared optics to achieve a ~0.9 mm diameter spot at the pupil (see **Table 1** for a list of all optical elements). Ophthalmic relay optics were optimized using a custom-designed murine eye model for spot-size performance across +/- 30 deg. retinal eccentricity for OCT, SLO, and laser lesioning sources. An additional polymethylmethacrylate (PMMA) zero-diopter meniscus lens was added to the air-corneal interface during optimization to model custom contact lenses used during imaging (**Figure 2**). Imaging resolution at the retina is limited by the lateral spot size of the 488 nm excitation for SLO, which is ~3 µm, and lateral spot size and coherence length of OCT, which are ~5 µm and 6.3 µm, respectively. While optical resolution *in vivo* was ultimately limited by aberrations in the mouse eye, these simulated spot sizes are comparable to previously published small-animal ophthalmic SLO and OCT imaging systems (9, 12).

**Table 1:** Parts list for key system components.

**Imaging Optics**	**Visible**	f_c_	Olympus	UPLFLN 10X2	**Detection Optics**	**Fluorescence**	f_c_	Thorlabs	AC254-030-A
		DM_1_	Semrock	LPD02-488RU-25			f_λ1_	Semrock	NF03-532E-25
		DM_2_	Chroma	ZT532dcrb			DM_4_	Semrock	FF553-SDi01
		f_c_	Thorlabs	T25FC-532			f_λ2_	Semrock	FF01-509/22-25
		f_s_	Thorlabs	AC254-400-A			f_λ3_	Semrock	FF01-632/148-25
		Thorlabs	AC254-100-A	PMT			Hamamatsu	H7422
		Thorlabs	LF1141-A	PM			Thorlabs	MPD249-G01
	**NIR**	f_c_	Thorlabs	F280APC-850		**OCT**	VPHG	Thorlabs	600lpmm
		f_s_	Thorlabs	AC254-400-B			f_obj_	Thorlabs	AC508-250-B
			Thorlabs	AC254-100-B				Thorlabs	AC508-300-B
			Thorlabs	LF1141-B				Newport	KBC073AR.16
	**Shared**	DM_3_	Edmund	67-077				Thorlabs	AC508-300-B
		f_oph_	Thorlabs	AC254-050-AB				Thorlabs	AC508-250-B
			Thorlabs	AC254-030-AB			CMOS	Basler	spL4096-140km
		f_cont_	Custom	F=-400 mm (Acrylic					

**Figure 3:**
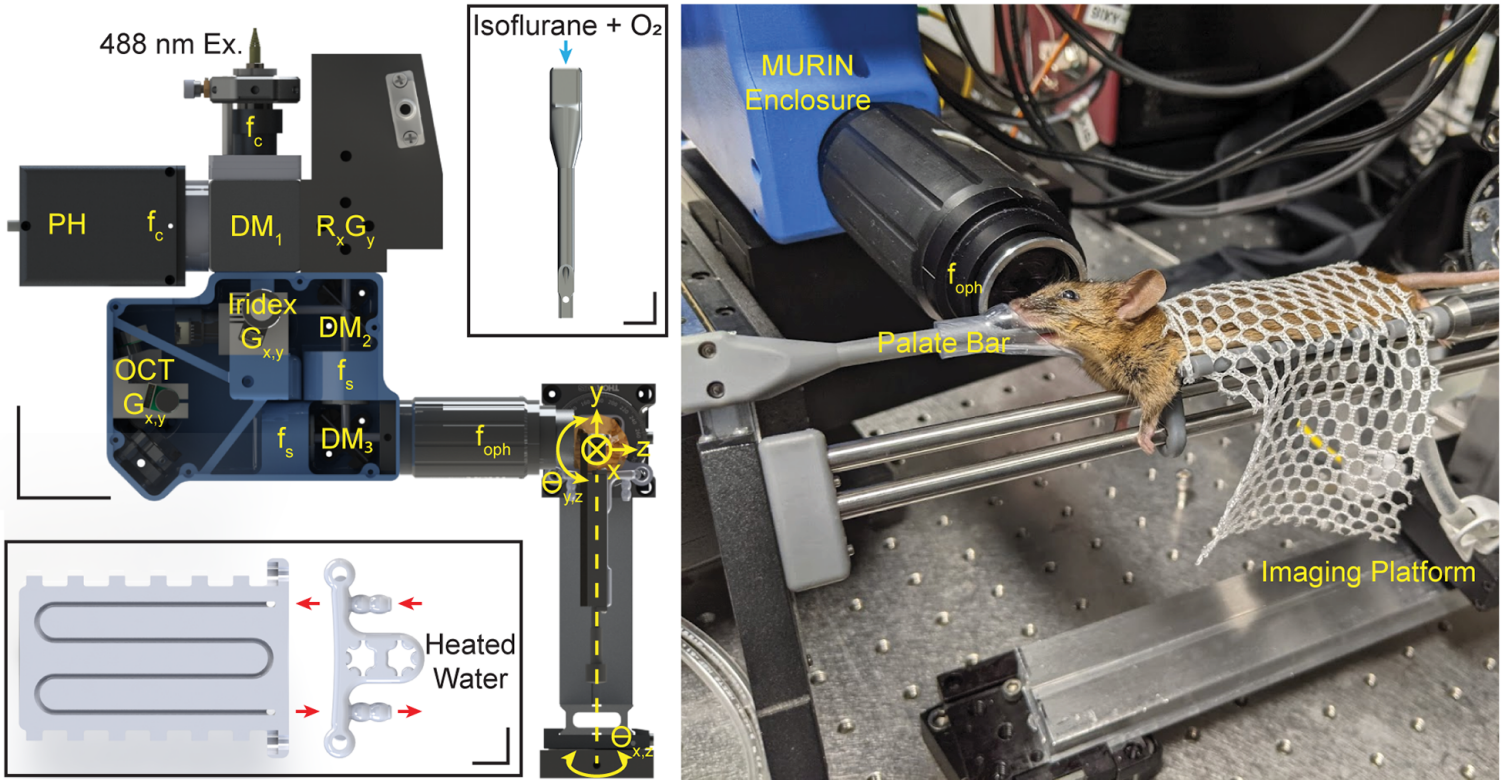
Mechanical layout for MURIN showing optics and optomechanics in the custom-designed mechanical enclosure and 5-axis imaging platform. DM: dichroic mirrors; f: collimating, objective, and scan lens; G: galvanometers; R: resonant scanner. Scale bar: 50 mm. Insets show custom-designed palate bar and water-heated imaging bed. Scale bars: 10 mm. Photograph shows animal during an imaging experiment with head positioned using the palate bar with nose cone and the body resting on the imaging bed attached to the 5-axis imaging platform.

**Figure 4:**
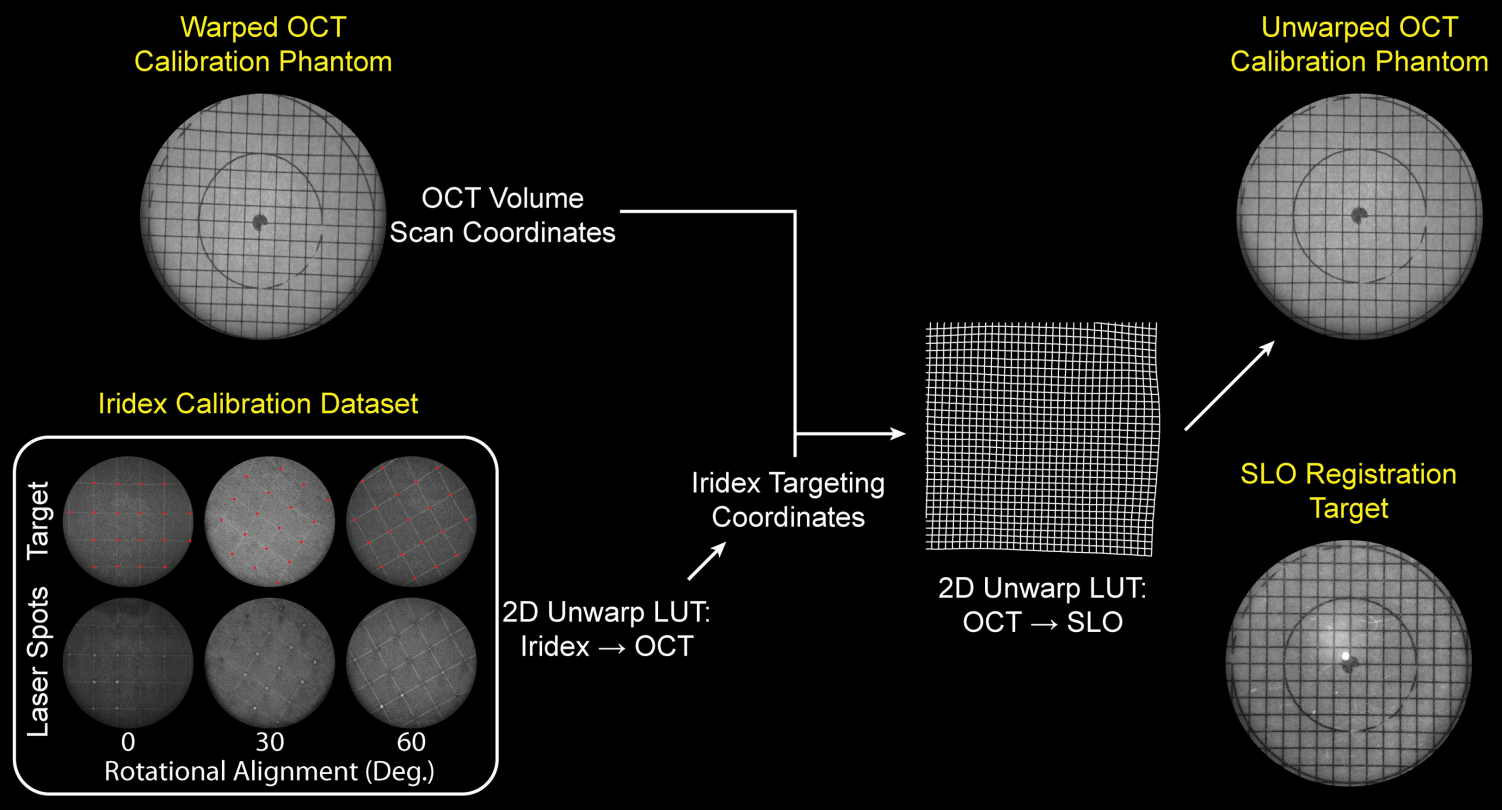
Laser lesion aiming calibration and OCT-SLO image co-registration flowchart. Inset shows target and actual lesion locations on grid calibration target used to calibrate laser lesion aiming locations to OCT FOV. Grid calibration target is imaged using both OCT and SLO and a 2-dimensional unwarp mask is computed to co-register OCT volumes to SLO images in post-processing.

All optics, optomechanics, and scanning elements were aligned in a custom-designed rapid-prototyped enclosure with the final ophthalmic lens on a manually tunable zoom element to enable dynamic focus adjust and retinal image flattening during data acquisition (**Figure 3**). Co-registration of the laser lesioning and OCT fields-of-view was performed by delivering laser pulses to a paper grid target at different rotational orientations. Offsets between target and lesion positions on grids were subsequently measured on OCT and used to extract a 2-dimensional polynomial voltage-to-position mapping for precise targeting (**Figure 4**). The same grid target was also imaged using both OCT and SLO and to compute a 2-dimensional unwarp transform (13) to co-register all MURIN modalities in post-processing (**Figure 4**).

### Animal imaging

*In vivo* retinal imaging was performed under an animal protocol approved by Vanderbilt University Medical Center. Mice were anesthetized using 2.4% Isoflurane, dilated with 1% Tropicamide drops, and fitted with our custom zero-diopter contact lens. Lubricant eye drops (GenTeal, Alcon) were periodically applied during imaging to index-couple the contact lens to the eye. Transgenic Müller glia-tdTomato reporter mice (*Rlbp1:CreER; Rosa^ai14^*, 5 animals, 10 eyes) and microglia-GFP/Müller glia-tdTomato reporter mice (*Cx3cr1^GFP^; Rlbp1:CreER; Rosa^ai14^*, 9 animals, 15 eyes) were used as models to visualize cellular changes in the retina after laser lesion delivery. All animals were adults, between 4 and 8 months of age. OCT and SLO images were acquired immediately before and after laser lesioning and again at 7- or 21-days post-laser. Average imaging time was <10 min per eye. Histological samples were taken immediately after lesioning, at 7- or 21-days post-laser. Dissected posterior eyecups were fixed in 4% paraformaldehyde overnight, cryoprotected in 20% sucrose, and stored at -80°C C in OCT (Sakura Fintech). 15 &mu;m cryosections were prepared for histology and imaged on a Nikon E600 epifluorescence microscope using a 20x plan fluor objective and a Nikon DS-Qi2 camera.

OCT images were acquired at 70 kHz line-rate (Basler, spL4096-140km). Dual channel SLO fluorescence images were acquired at 7.8 frames-per-second (1024 x 1024 pix.) on photomultiplier tubes (Hamamatsu, H7422-20) through a 100 µm diameter confocal pinhole. The pinhole diameter corresponded to ~2 Airy units and was optimized for fluorescence collection efficiency instead of confocal sectioning. SLO and OCT were designed to be parfocal and was adjusted to maximize GFP and tdTomato fluorescence on SLO. OCT, SLO, and laser lesion scanning are software-controlled independently and can be performed simultaneously for multimodality imaging of retinal injury. A custom palate bar and nose cone were used to reduce bulk motion, and animals were aligned using a custom 5-axis degree-of-freedom platform. The imaging platform also included a custom-designed water-heated bed to maintain animal body temperature while under anesthesia (**Figure 3**).

### Image-guided laser lesioning

Custom LabView control software was developed to steer the photocoagulation laser and automatically deliver laser pulses to targets-of-interest. Extended area laser lesion patches were created by delineating a treatment area (e.g., avoiding retinal vessels) on en face OCT projections and setting parameters for lesion spacing within that treatment area. Laser parameters, including laser pulse duration, power, number of repeat pulses, and interval (i.e., delay between repeat pulses) can also be set for all lesions.

### Image-processing

OCT images were sampled with 4096 x 500 x 500 pix. (spectrum x lines x frames) and 5 repeated B-scans at each position for a total of 2500 frames-per-volume and SLO images were sampled with 1024 x 1024 pix. Repeated OCT B-scans were averaged and en face views were obtained by depth-averaging between the outer plexiform layer and retinal pigmented epithelium (RPE) in a digitally flattened volume. SLO datasets consisted of 200 frames acquired at 7.8 Hz. In post-processing, SLO frames with significant respiratory motion artifacts were removed and the remaining frames were co-registered and averaged for improved signal-to-noise ratio (14, 15).

Co-registered and averaged 5-repeated frames were denoised using self-fusion to enhance signal-to-noise-ratio (14). Manually annotated OCT cross-sections were used as ground truths to train a U-Net convolutional neural network. The network was designed and implemented in PyTorch based on a multi-scale U-Net architecture (16). During hyperparameter tuning, the data was split into 80% (32 volumes) for training and 20% (8 volumes) for validation. The cross-entropy loss and Dice score were used to compare the performance of the network for different hyperparameters. The network was trained up to 50 epochs. After the hyperparameters were defined, 10-fold and 5-fold cross-validation strategies were followed to estimate the performance of the model. The Dice coefficient was used to validate automated segmentation performance.

## Results

**Figure 5:**
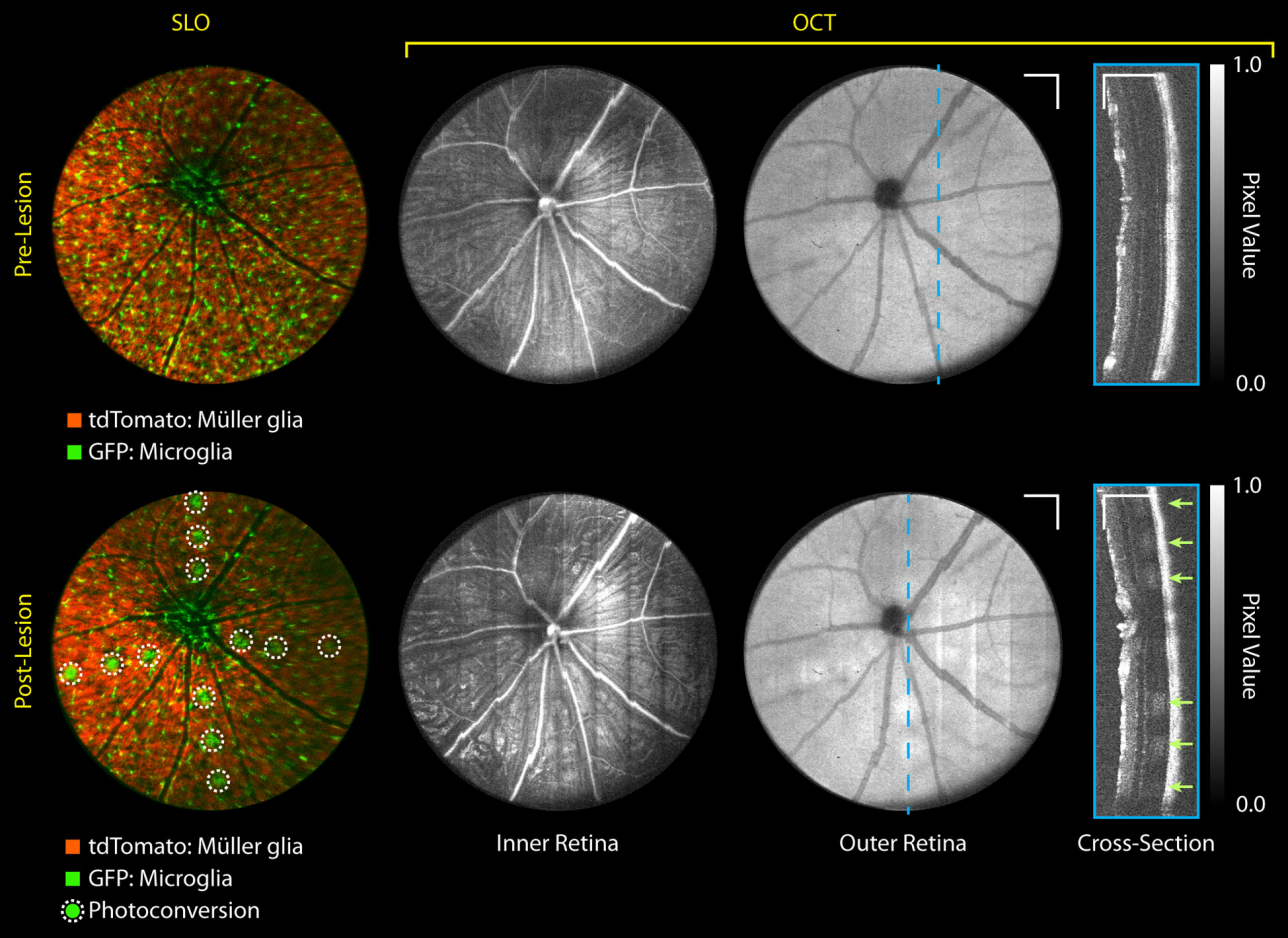
MURIN retinal imaging pre- and immediately post-laser lesioning. 3 lesions were placed in each of the 4 quadrants (nasal, temporal, superior, inferior) with a single 6 mW 200 ms duration laser pulse (arrows). These low-severity lesions show small increases in OCT scattering only on outer retinal projections and depth cross-sections, but SLO shows high-contrast fluorescence photoconversion of tdTomato. Grayscale contrast in both OCT volumes were matched using speckle background to visualize relative scattering increases at the lesion. Intensity non-uniformity in OCT inner retinal projects is a volume projection artifact and does not reflect differences in image quality across the FOV. Scale bar: 250 µm. Colorbar: Normalized pixel intensity values.

**Figure 5** shows SLO and OCT images immediately before and after laser lesioning in microglia-GFP/Müller glia-tdTomato mice. Here, low-severity lesions were created using a single 200 ms duration laser pulse with 6 mW at the pupil. Lesions were aligned nasal-temporal and inferior-superior between vessels with 3 lesions per quadrant. SLO fluorescence shows high-contrast photoconversion of tdTomato fluorescence from the red PMT channel (558-706 nm) to the green PMT channel (498-520 nm). These low-severity lesions show minimal increases in OCT scattering in the outer retina above the retinal pigment epithelium (RPE) from preferential absorption of the 532 nm photocoagulation laser by RPE melanin. While fluorescence photoconversion has been previously described (17, 18), this proof-of-concept data shows it might provide higher sensitivity for quantifying retinal photodamage as compared to OCT, especially for low-severity lesions.

**Figure 6:**
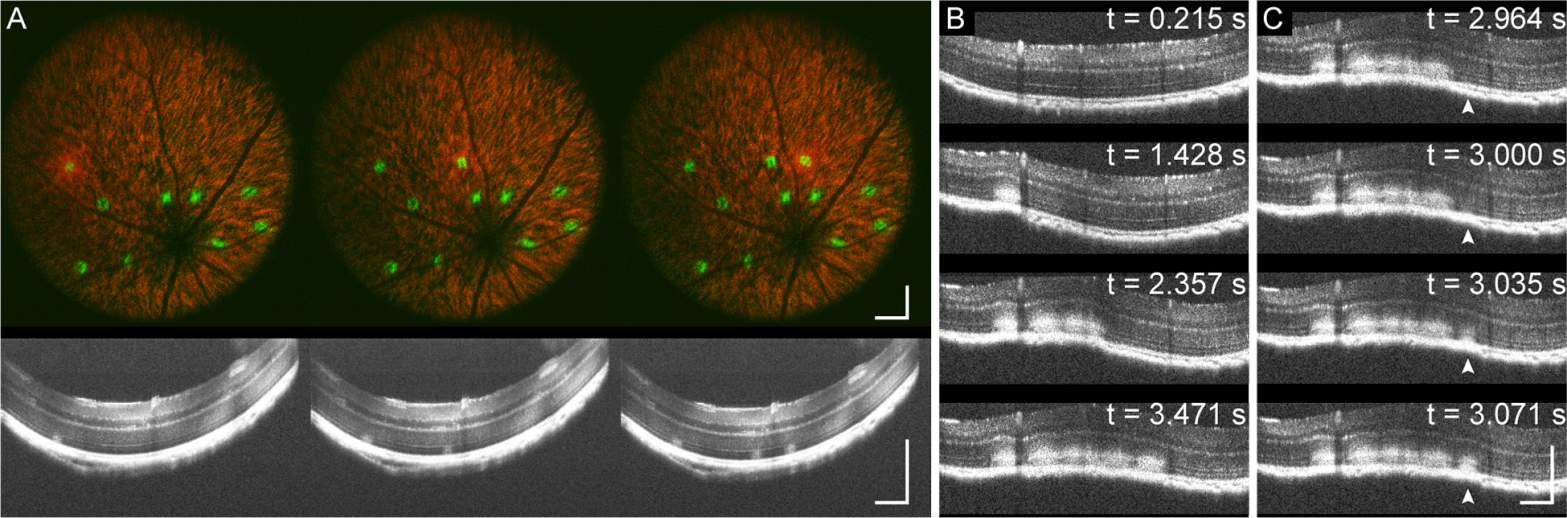
MURIN imaging of laser lesion dynamics. **(A)** Video-rate imaging of photodamage with 4 successive 6 mW 200 ms duration laser pulses at each location shows increasing fluorescence photoconversion on SLO and scattering contrast on OCT cross-sections (**Supplemental Video 1**). **(B)** Higher severity damage with 5 successive 20 mW 300 ms duration laser pulses show deformation of the retina resulting from local heating (**Supplemental Video 2**). **(C)** Images of individual pulses show accumulation of injury at the RPE propagating into the inner retina (arrow). Scale bar: 250 µm.

Real-time MURIN imaging concurrent with laser lesioning also allows us to visualize lesion formation dynamics and any corresponding changes in retinal morphology. Unsurprisingly, photodamage severity accumulates with repeat laser pulses at the same location. **Figure 6A** shows SLO and OCT cross-sections of lesion formation acquired at video-rates for 4 successive 6 mW 200 ms duration laser pulses (**Supplemental Video 1**). With each laser pulse, we observe increasing fluorescence photoconversion on SLO and scattering contrast on OCT. At even higher photodamage severity with 5 successive 20 mW 300 ms duration laser pulses, we observe bowing of the retina on OCT cross-sections from local heating and expansion of the underlying retinal structure (**Figure 6B** and **Supplemental Video 2**) (19, 20). When looking at changes in OCT contrast for each pulse (arrow, **Figure 6C**, we observe propagation of scattering changes at the lesion from the RPE into the uter retina accompanying flattening of the retinal curvature. Here, MURIN imaging is performed in Müller glia-tdTomato mice without microglia-GFP contrast as a negative control to confirm green fluorescence shown in **Figure 5** was indeed from tdTomato fluorescence photoconversion.

**Figure 7:**
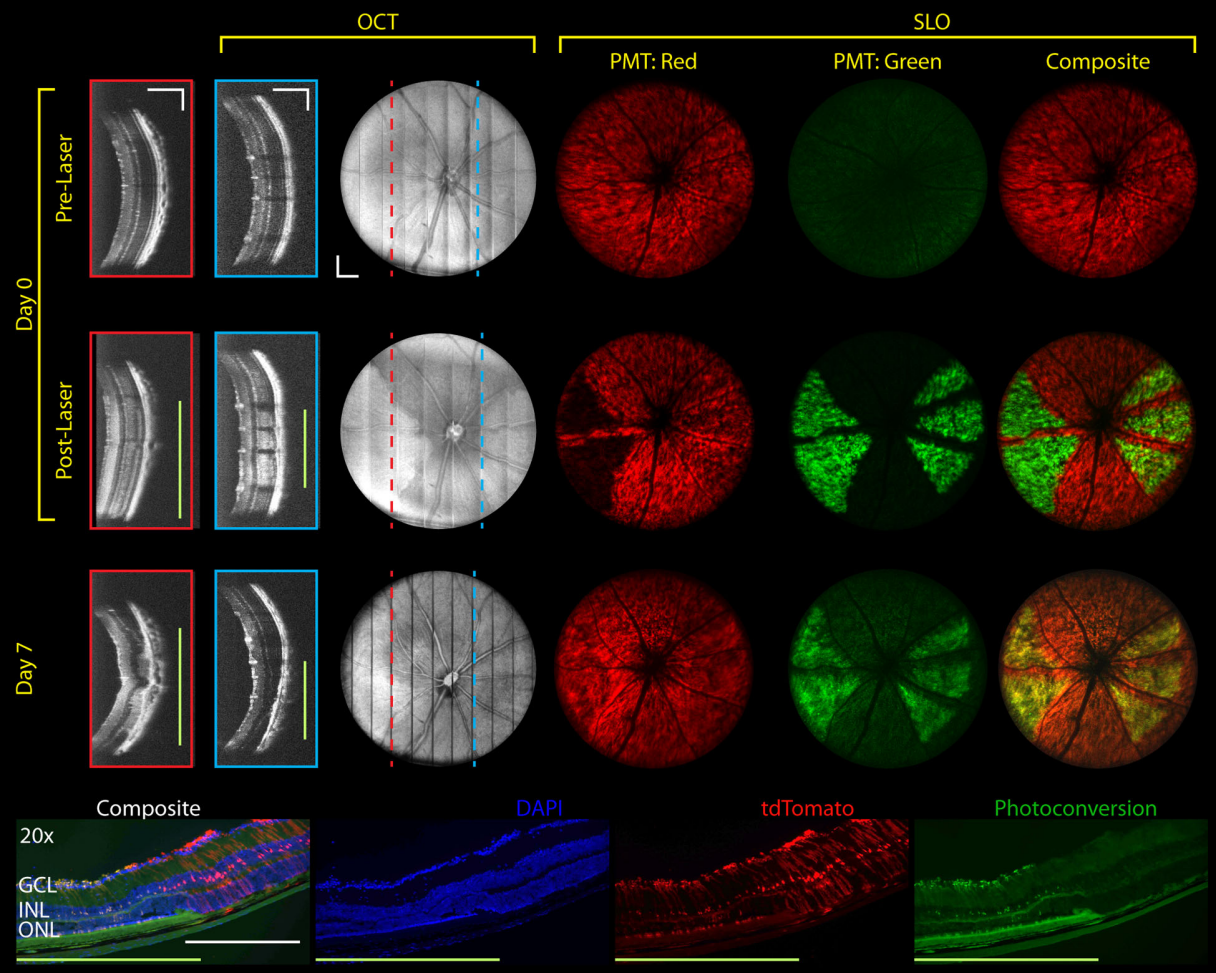
Longitudinal MURIN imaging of photodamage patches. Patches were scanned using 50% spot-size overlap 6 mW 200 ms duration laser pulses. Higher and lower injury levels (left and right of the ONH) were created using 4 and 1 laser pulses per location, respectively. OCT images show cross-sections in higher and lower injury locations (red and blue dotted lines and boxes, respectively). On SLO, higher severity patches showed more complete fluorescence photoconversion and retinal deformation (focal shift on **Supplemental Video 3**). Photoconversion is confirmed on representative histology (bottom) and persists at 7-days post-laser lesioning. Green bars indicate photodamage regions. Scale bar: 250 µm.

Spatial changes in retinal morphology on OCT and fluorescence on SLO are more prominently visualized when creating patches of retinal photodamage (**Figure 7** and **Supplemental Video 3**). Here, laser lesion patches between vessels were delineated with high and low-severity levels located on the left and right side of the optic nerve head (ONH), respectively. Patches were created by automatically raster-scanning the photocoagulation laser by 50% spot-size overlap across the delineated laser lesion patch with 6 mW 200 ms duration laser pulses. High and lower severity patches were created using 4 repeated and single laser pulses per spatial location. While en face OCT volume projections do not show significant changes in scattering contrast, lesions and corresponding retinal layer changes at 7-days post-laser lesioning are readily visible on OCT cross-sections. Fluorescence photoconversion is clearly visible immediately after laser lesioning with almost complete photoconversion of the tdTomato contrast in the high-severity patches. While the shifted green-fluorescence remains visible 7-days post-laser lesioning, additional tdTomato fluorescence also returns to laser lesion patches. Widefield epifluorescence of representative histology taken immediately after laser lesioning (**Figure 7** bottom) confirms fluorescence shift from red to yellow in the laser lesion patch as well as increased green fluorescence at the RPE. Changes in retinal curvature from local heating is also observed on SLO as a focal plane shift in the fluorescence signal.

**Figure 8:**
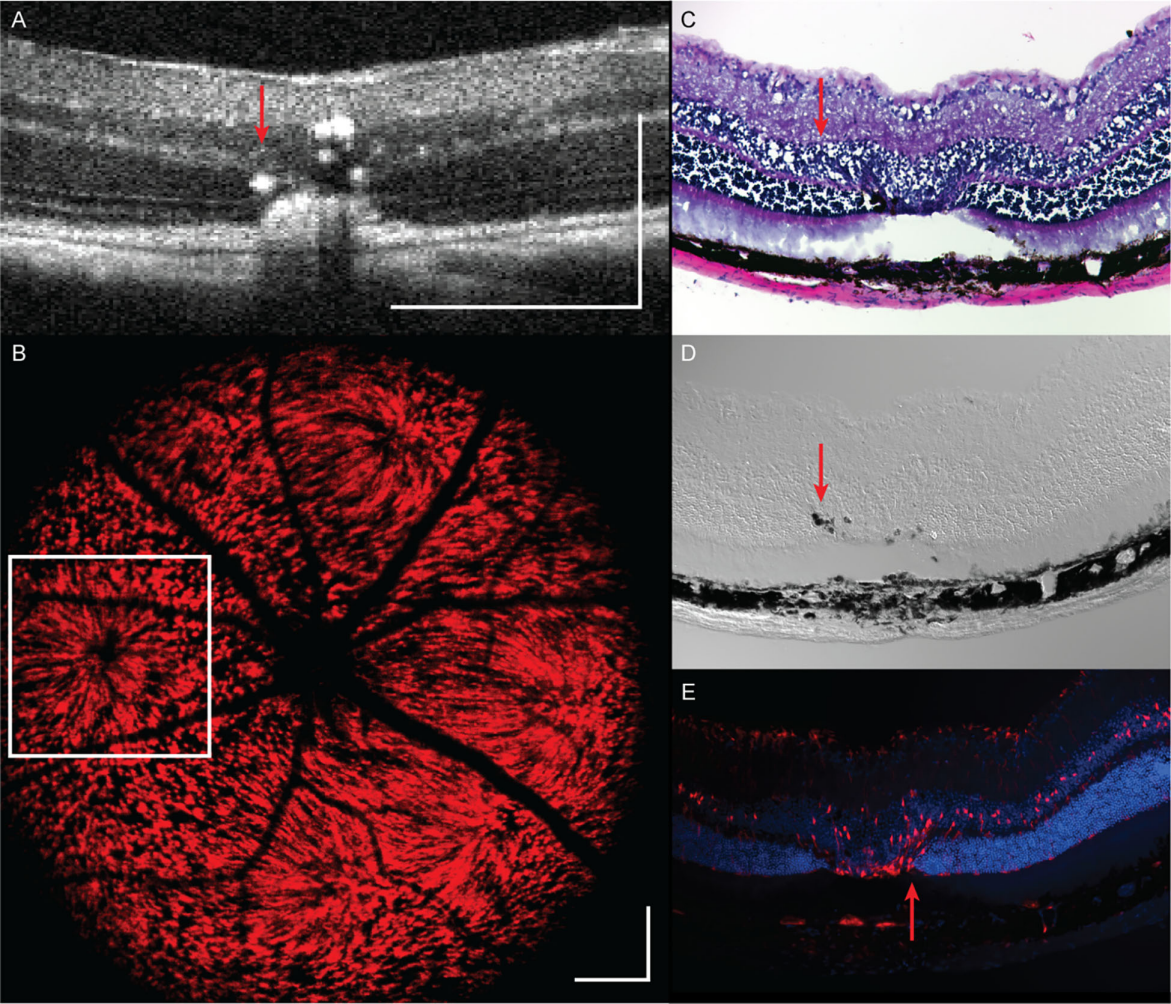
Retinal structural changes at 21 days following a single 20 mW, 300 ms duration laser pulse as assessed by **(A)** OCT, **(B)** SLO, **(C)** H&amp;E histology, **(D)** DIC microscopy, and **(E)** epifluorescence. Scale bar: 250 µm. Red arrow: hyper-pigmented cells and debris within laser lesions.

Significant morphological changes are visible on MURIN after high-severity photodamage. Laser lesions from single 20 mW 300 ms duration laser pulses were imaged at 21-days post-laser lesioning (**Figure 8**). OCT cross-sections show the spatial extent of the lesions contract over time from diffusion areas of increased scattering to granular scatterers and corresponding SLO images show a radial pattern surrounding severe focal lesions, which may be a result of a change in Müller cell shape or orientation in response to injury. The inner plexiform layer is distorted and increased RPE thickness and scattering are observed, all of which are confirmed on corresponding hematoxylin and eosin (H&amp;E) histology and differential interference contrast (DIC) microscopy. H&amp;E histology shows a clear loss of cells within the outer nuclear and photoreceptor layers and a retinal detachment at the lesion. However, we believe the detachment occurred during tissue processing since the OCT image (taken on the same day as tissue dissection) shows no sign of detachment. The OCT and DIC images reveal individual scatterers within the lesion volume, which may be melanocytes that have migrated from the RPE layers (red arrows). Similar scattering features on OCT and hyper-pigmented cells and debris within laser lesions have also been previously described (21). Widefield epifluorescence of cryosection shows Müller glia expressing tdTomato in red and DAPI-stained nuclei in blue. The Müller glia can be seen oriented radially around the lesion (red arrow) which corresponds well to the radial projections visualized on SLO (white box).

**Figure 9:**
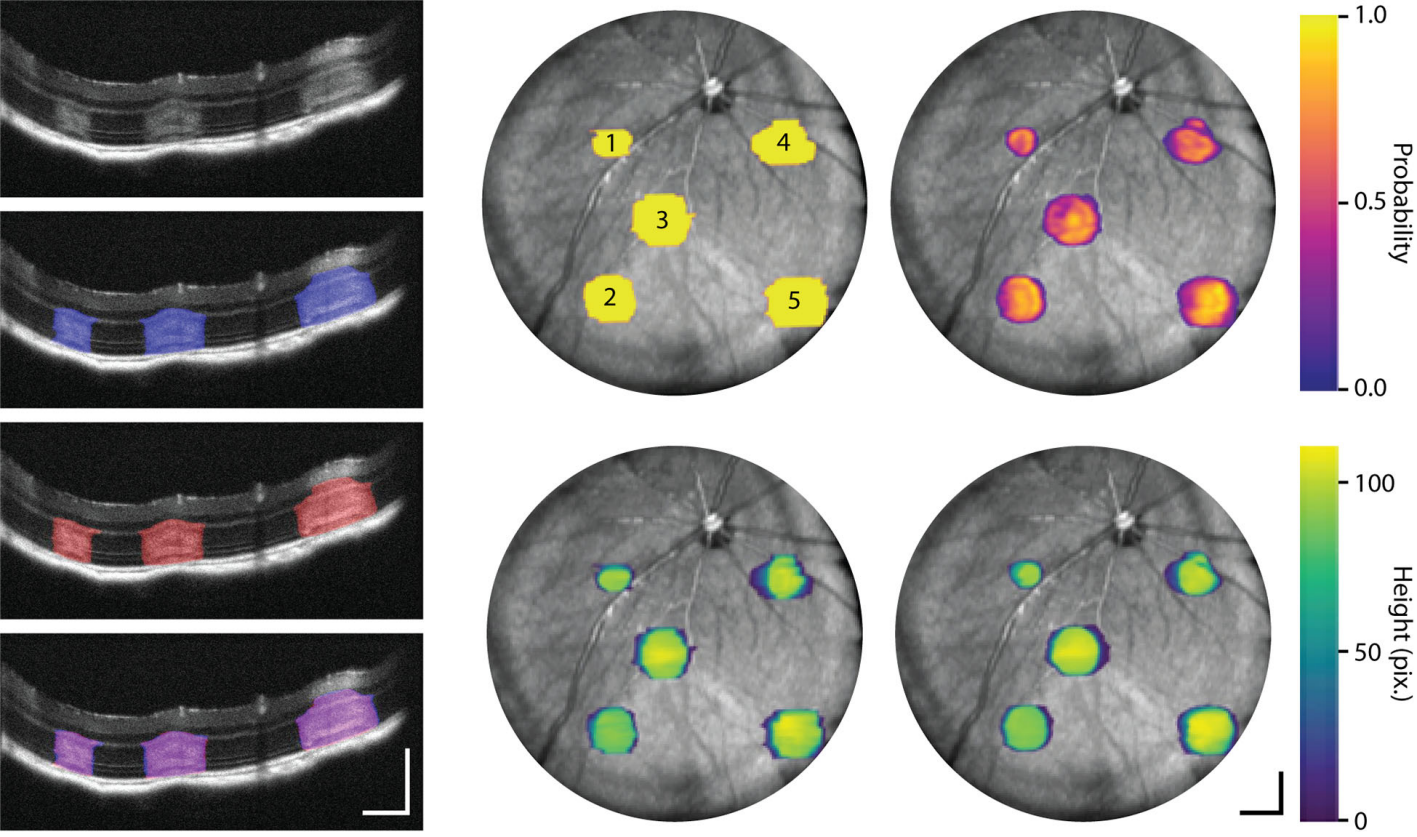
Machine-learning-based segmentation of laser lesions on OCT. Lesions were created with 4 repeated 100 ms pulses with 10, 20, 30, 40, 50 mW power (labeled 1-5, respectively). OCT cross-sections of laser lesions with manual (blue), automated (red), and manual/automated overlap (purple) labeling (**Supplemental Video 4**). En face OCT projections show neural network segmentation (right column) probability maps indicating level of certainty in delineating lesions and OCT thickness of each lesion as compared to the corresponding manually labeled volumes (middle column). Scale bar: 250 µm.

While fluorescence photoconversion contrast may provide high sensitivity feedback on the location of photodamage, it does not provide three-dimensional information about laser lesion size. While OCT volumes clearly show changes in scattering contrast resulting from laser lesioning, quantification requires manual labeling of lesions on successive OCT cross-sections, which is cumbersome and error-prone. Using our custom-trained neural network, we demonstrate proof-of-concept automatic laser lesion segmentation on OCT volumes (**Figure 9** and **Supplemental Video 4**). Here, lesion size and severity were titrated using 4 repeated 100 ms pulses with 10, 20, 30, 40, 50 mW power (labeled 1-5, respectively) to demonstrate robust automated segmentation across different lesion sizes and appearance on OCT cross-sections. Comparison of manual and automatic lesion segmentation on OCT cross-sections shows a high degree of spatial co-registration. En face volume projections of these lesions show smoother segmentation contours in automatically segmented lesions, highlighting more robust sensitivity to scattering changes in lesions as compared to manual visualization.

## Discussions

We have demonstrated MURIN as a unique imaging platform that enables combined SLO and OCT imaging with an integrated image-guided laser lesioning module. This technology has clear benefits over existing multimodal imaging and laser lesioning systems by enabling simultaneous multimodal imaging, independent and precise control of Iridex laser pulse parameters and patterns, and real-time OCT and SLO visualization of lesion formation. Implementation of image-guided laser lesion mapping allowed us to avoid regions-of-interest, such as large vessels or the optic nerve head and may be used to generate pan-retinal injuries to model human pathologies (e.g., geographic atrophy) while avoiding potential retinal hemorrhage. While using en face OCT projections to map laser lesion locations does require a pre-laser OCT volume acquisition and ~1 minute delay between mapping and lesioning, we observed no errors from motion when using our custom-designed imaging platform and palate bar.

In this study, transient (~30-100 ms) respiratory motion was observed at ~1-2 Hz and more prominent in the axial direction (<50 µm lateral and <200 µm axial displacement). Lateral motion is expectedly minimal because lateral shifts of the beam position on the pupil result in small angular shifts to are manifested as tilts in the OCT depth cross-sections instead of en face lateral translation. While motion artifacts from respiratory motion during laser lesioning may introduce variability in both the amount of energy delivered to specific locations on the retina and corresponding retinal injury severity, we did not observe or exhaustively investigate these effects in this proof-of-concept imaging study. However, these effects can potentially be mitigated by using the respiratory motion to gate laser lesioning to ensure laser pulses are only delivered between respiratory cycles.

We performed observational studies on the effects of pulse duration, power, and number of pulses on lesion severity and laser lesion patches using MURIN (**Figures 5**–**9**). While on average, laser lesion size corresponded directly with the amount of laser lesion energy delivered, we do note that increased optical aberrations and reduced spot size performance at the edges of our FOV (**Figure 2**) and potential ocular opacities in the animal may significantly the robustness of our laser injury system. In this study, we highlight two potential methods for directly quantifying actual injury severity at the retina that is decoupled from optical aberrations and losses. We report the first observation, to our knowledge, of spectral shift of tdTomato fluorescence as a result of photoconversion in *in vivo* retina (**Figures 5**–**7**). Quantification of photoconversion (i.e., ratio of tdTomato and spectrally shifted fluorescence signal) may provide a high-sensitivity method of quantifying local injury. In non-transgenic animals, we demonstrated automated machine-learning based laser lesion segmentation using OCT. This segmentation approach may be used to quantify lesion volumes in real-time and assess retinal injury severity. While additional work remains to be done to validate the reproducibility of these methods, we believe the real-time multimodality imaging capabilities of MURIN can be leveraged to titrate retinal injury severity *in vivo* in real-time.

## Data availability statement

The raw data supporting the conclusions of this article will be made available by the authors, without undue reservation.

## Ethics statement

The animal study was reviewed and approved by Vanderbilt University Medical Center.

## Author contributions

JR-J: algorithms development and interpretation of data. SN: system design, optimization, and calibration. JJ: study design, data collection, and interpretation of data. JM: system design, study design, and data collection. GR: data analysis and interpretation of data. EL: study design, interpretation of data, and review of manuscript. YT: study design, system design, data collection, data analysis, and review of manuscript. All authors contributed to the article and approved the submitted version.

## Supplementary material

Video 1 Fluorescence photoconversion and increased OCT scattering from photodamage.

Video 2 Retinal deformation from photodamage.

Video 3 Fluorescence photoconversion and retinal deformation in photodamage patches.

Video 4 Machine-learning-based segmentation of laser lesions on OCT.
